# The experiences of dealing with consequences of an avalanche – surviving soldiers’ perspectives

**DOI:** 10.1080/17482631.2019.1689066

**Published:** 2019-11-12

**Authors:** Lars-Petter Bakker, Siren Eriksen, Jon Gerhard Reichelt, Ellen Karine Grov

**Affiliations:** a Norwegian Armed Forces Joint Medical Services, Institute of Military Psychiatry, Oslo, Norway; b Faculty of Health Studies, Norwegian National Advisory Unit on Ageing and Health, Vestfold Hospital Trust, Tønsberg, Norway, and VID Specialized university, Oslo, Norway; c Department of Nursing and Health Promotion, Faculty of Health Sciences, Oslo Metropolitan University, Oslo, Norway

**Keywords:** Health, avalanche, trauma, coping strategies, daily life, qualitative interviews, content analysis, well-being

## Abstract

**Purpose**: The aim of the study was to explore and describe experiences of daily life after having experienced an avalanche three decades ago.

**Method**: This paper presents a qualitative study of 12 male survivors of an avalanche during their military service, interviewed 30 years post-disaster.

**Findings**: A comprehensive understanding of the categories led to the latent theme “Finding my own way of managing and dealing with life”. Findings revealed three categories describing experiences of daily living: (i) A comfortable life; (ii) A challenging, yet accomplished life; (iii) A demanding life. The first category represents a greater degree of using adaptive coping strategies for managing everyday life compared to the other two categories. The third category represents the group having the most challenging consequences. Among the three, the latter category conveys the most maladaptive coping strategies.

**Conclusions**:The participants had different experiences with regards to their health and how they coped with their everyday life after the avalanche disaster. Insights into coping strategies may provide a guide for appropriate interventions for survivors dealing with traumatic events.

## Introduction

Every year disasters affect millions of people around the world (approximately 141 million victims in 2014) (Guha-Sapir, Hoyois, & Below, 2015)), and there is, on average, one reported disaster every day worldwide (Goldmann & Galea, 2014; Guha-Sapir et al., 2015; North, 2016). Studies have reported that 10–19% of adults will experience a type of disaster in their lifetime (Darves‐Bornoz et al., 2008; Goldmann & Galea, 2014; Kessler, Sonnega, & Bromet et al., 1995). Mainly, the research literature defines disasters as traumatic events (TEs) that are collectively experienced, time-delimited, and have an acute onset (McFarlane & Norris, 2006). Further, in psychology the term TE seems to be used to describe a catastrophic and severely distressing event, e.g., as it is done in the Diagnostic and Statistical Manual of Mental Disorders fifth edition (DSM-5) (American Psychiatric, Association, 2013). Furthermore, in literature, TEs as disasters often are frequently categorized into three types (Goldmann & Galea, 2014; McFarlane & Norris, 2006): (i) man-made disasters, (ii) non-intentional technological disasters, and (iii) natural disasters (Goldmann & Galea, 2014; McFarlane & Norris, 2006). North (2016) writes in her review that most knowledge of TEs has, in a historical perspective, been gained by research on nondisaster traumas. However, the exposure to TEs as disasters are a major worldwide problem, and studies of disasters are associated with a broad variety of negative mental health (psychopathology) and physical health effects (Afari et al., 2014; Ásgeirsdóttir et al., 2018; Benjet et al., 2016; Bøe, Holgersen, & Holen, 2011; Bromet et al., 2017; Bromet, Karam, & Koenen et al., 2018; Galea, Nandi, & Vlahov, 2005; Goldmann & Galea, 2014; Kessler et al., 2017, Koenen et al., 2017; Lassemo, Sandanger, Nygård, & Sørgaard, 2017; Lawrence, Lin, & Lipton et al., 2019; Neria, Galea, & Norris, 2009; Neria, Nandi, & Galea, 2008; Norris, Friedman, & Watson et al., 2002; North, 2016; Pacella, Hruska, & Delahanty, 2013; Thordardottir et al., 2015; Yzermans, van den Berg, & Dirkzwager, 2009). A recent systematic review (Steinert, Hofman, Leichsenring, & Kruse, 2015) of the course of PTSD in naturalistic long-term studies claims that PTSD presumably is the core psychopathology following trauma (Breslau, Chase, & Anthony, 2002; Neria et al., 2008; Steinert et al., 2015). Although, studies of TEs have shown that the majority of victims do not develop a mental health disorder (Breslau et al., 1998; Norris, Tracy, & Galea, 2009), and over the past few decades, interest in resilient and growth patterns or trajectories has increased due to the fact that most people exposed to TEs cope well post-disaster (Bonanno, 2004; Bonanno, Galea, Bucciarelli, & Vlahov, 2006, Tedeschi & Calhoun, 2004).

A large number of studies aim to find risk factors that can predict different adverse health outcomes after disasters and TEs (Brewin, Andrews, & Valentine et al., 2000; Galea et al., 2005; Neria et al., 2008; Norris et al., 2002; Ozer, Best, Lipsey, & Weiss, 2003, Rubonis, Bickman, & Steinberg, 1991; Shalev, Tuval-Mashiach, & Hadar, 2004). However, description of factors that may identify population or individuals at risk of developing PTSD, are the most common approaches in literature to predict adverse health outcome post-disaster. Further, risk factors can be divided into three groups, respectively risk factors that may predict and increase vulnerability to psychopathology, (i) before (pre) (e.g., prior mental health problems, gender, age), (ii) during (peri) (e.g., the degree or severity of the exposure and proximity) and (iii) after (post) trauma (e.g., stressors as job loss, property damage, reduction in and low level of social support) (Goldmann & Galea, 2014).

Previous studies have found significantly more social and occupational functioning problems in people with psychopathology post-disasters than those without psychopathology in the initial days and months post-disaster (North, 2016; North & Oliver, 2013; North, Pffferbaum, Kawasaki, Lee, & Spitznagel, 2011). However, a study by North et al. (2011) found, during a time frame of 7-years post-disaster that functioning problems decline over time and largely resolved, even among individuals with PTSD still experiencing symptoms (North, 2016; North & Oliver, 2013; North et al., 2011). North (2016) suggests that even though psychopathology symptoms continued post-disaster, individuals managed to find ways to cope in their everyday life and move on, regardless if they had PTSD-symptoms or not (North, 2016). Several patterns have been reported in the literature regarding the course of PTSD-symptoms, and trauma-related psychopathology, e.g., U-shaped pattern (Macleod, 1994; Port, Engdahl, & Frazier, 2001), chronic pattern (Bonanno, 2004; Norris et al., 2009), delayed pattern (Bonanno, 2004), recovery pattern (Bonanno, 2004; Norris et al., 2009), resilience pattern (Bonanno, 2004; Norris et al., 2009), and resistance pattern (Norris et al., 2009), see Appendix 1.

There are many different ways to cope with everyday life and adverse life events after experiencing stressful situations and TEs—both in short and long term. However, in literature mainly coping is considered as a regulatory process that can reduce the negative feelings resulting from stressful situations as TEs (Afshar et al., 2015; Compas, Connor-Smith, Saltzman, Thomsen, & Wadsworth, 2001). Lazarus & Folkman (1984) defined coping styles as the behavioural and cognitive efforts (e.g., like the changing of action and thoughts (Lazarus, 1991, 1999)) to manage internal and external stressors. Another definition refers to coping strategies as psychological and behavioural efforts to tolerate, overcome, or reduce the impact of stressful events (Carver, 1997). Further, some researchers emphasize that coping is a dynamic process that fluctuates over time in response to changing appraisals and demands of the situation (Afshar et al., 2015; Diehl et al., 2014; Moos, Holahan, & Beutler, 2003). Furthermore, Rice and Liu (2016) argue that coping is actions taken to deal with any type of stressor, regardless large or small, or occurring in daily or in the long run.

In research literature on stress and coping, there are two major conceptual distinctions; (i) emotion- and problem-focused strategies (Carver, Scheier, & Geen, 1994; Folkman, Lazarus, & Hogan, 1985) and (ii) avoidance and approach strategies (Roth & Cohen, 1986; Snyder, 2001). On the basis of the theory of stress and coping, it is relevant to assume that different coping strategies (i.e., emotion, problem, avoidance and approach strategies) are used to manage stressful experiences such as avalanches. Most of the current coping-strategy literature relates coping to problem solving (e.g., active planning, specific behaviour to overcome the problem) and active emotional strategies (e.g., cognitively reframing the problem, humour) to positive psychological adjustment (Bartone et al., 2015; Bei et al., 2013; Cherry et al., 2017, Littleton, Horsley, John, & Nelson, 2007; Penley, Tomaka, & Wiebe, 2002; Schnider, Elhai, & Gray, 2007; Zimmer-Gembeck & Skinner, 2008). On the other hand, avoidant emotional coping strategies are viewed as more maladaptive coping strategies and may interfere negatively with mental health (Bartone et al., 2015; Bei et al., 2013; Cherry et al., 2017; Littleton et al., 2007; Penley et al., 2002; Schnider et al., 2007; Zimmer-Gembeck & Skinner, 2008).

In our discussion section we will consider our findings in the light of the theory of stress and coping strategies (Carver, 1997; Lazarus & Folkman, 1984; Skinner, Edge, Altman, & Sherwood, 2003), and the coping stratgies will mainly be interpreted through Skinner and colleagues’ (Skinner et al., 2003) five coping strategies: (i) problem solving, (ii) support seeking, (iii) avoidance, (iv) distraction, and (v) positive cognitive restructuring (see Appendix 2 for more details). Skinner and colleagues’ (Skinner et al., 2003) five coping strategies are integrated from analysing 100 coping-category systems proposed from the 1980s to 2000.

There might be several other theories and models in the literature that are related to the concept of coping, e.g., relation between personality and coping, and relation between resilience and coping. In our discussion section, we will also consider our findings in the light of some researchers using resilience theory. However, different coping strategies may be more appropriate for different people in different contexts and social environments. Therefore, it is important to see beyond individual factors that may promote coping and resilience and look into community factors as well. Several studies have discussed such factors beyond the individual level, such as within communities, families, or organizations (Docena, 2015; Kirmayer, Dandeneau, Marshall, Phillips, & Williamson, 2011; Kruse et al., 2017, Meredith, Sherbourne, & Gaillot et al., 2011; Rice & Liu, 2016; Southwick, Bonanno, Masten, Panter-Brick, & Yehuda, 2014), which will also be important to include and discuss in our paper. And the definition of resilience will be understood in this paper from the theoretical framework by Grotberg (1995, p. 7): “a universal capacity which allows a person, group or community to prevent, minimize or overcome the damaging effects of adversity”.

However, coping per se is not considered a characteristic of resilience (Rice & Liu, 2016). Further, Rice and Liu (2016) argue that resilience refers to dealing with commonplace circumstances, while coping refers to encounters in everyday life, as well as dealing with distress. Resilience is often defined as positive adaption to change, while not all coping strategies are necessarily helpful (Rice & Liu, 2016). Thus, while all persons use coping strategies, not everyone using coping strategies is considered resilient (Rice & Liu, 2016). Further, Rice and Liu (2016) claim that resilience refers to the result of adaptive coping strategies following major tragic events.

Quantitative studies on coping strategies have reported that coping strategies interpreted as adaptive, particularly problem solving and support seeking, are approaches found to contribute to better and healthier functioning (Cherry et al., 2017; Littleton et al., 2007; Xu & He, 2012; Zimmer-Gembeck & Skinner, 2008), and have a positive effect on mental health symptoms (Xu & He, 2012). Several qualitative studies after natural disasters are also consistent with these findings (Ekanayake, Prince, Sumathipala, Siribaddana, & Morgan, 2013, Ibañez, Buck, Khatchikian, & Norris, 2004; Rajkumar, Premkumar, & Tharyan, 2008), even though qualitative studies after natural disasters are rare. These qualitative studies indicated that the most cited adaptive coping styles were support seeking, problem solving, and seeking meaning (Ekanayake et al., 2013; Ibañez et al., 2004; Rajkumar et al., 2008). These latter coping styles were also found to contribute to better and healthier functioning (Ekanayake et al., 2013; Ibañez et al., 2004; Rajkumar et al., 2008). On the other hand, previous quantitative and qualitative studies after natural disasters have shown that maladaptive coping styles as e.g., avoidance and distraction are the most cited maladaptive coping styles (Bartone et al., 2015; Ekanayake et al., 2013; Ibañez et al., 2004; Rajkumar et al., 2008). Such coping styles are associated with impaired functioning, psychological distress and poor health (Bei et al., 2013; Cherry et al., 2017; Littleton et al., 2007; Schnider et al., 2007; Zimmer-Gembeck & Skinner, 2008). Further, one recent qualitative study explores the role of mental toughness and lived experience of survivors of an earthquake with a subsequent avalanche (Swann, Crust, & Allen-Collinson, 2016). This study was conducted only a short time after the disaster and did not perform a follow-up of the role of mental toughness in a long-term perspective. To compare a short-term follow-up study like this with our long-term follow-up might be a limitation. However, this study is relevant for our study since the study sample is former military personnel who is presumed to have relatively high levels of mental toughness. Further, mental toughness is considered an important trait regarding coping with stress (Swann et al., 2016). However, the definitions of the term “toughness” are widely-differing. Nevertheless, one proposed definition is that mental toughness is an ability to cope with or handle pressure, stress or adversity (Goldberg, 1998, Gould, Hodge, Peterson, & Petlichkoff, 1987; Jones, 2002; Williams, 1988). The study by Swann, Crust, and Allen-Collinson (Swann et al., 2016) found that mental toughness has a positive role on coping during trauma and shortly post-disaster. The mentally tough survivors in this study reported that they were less likely to dwell over the disaster in the immediate aftermath, and they placed emotions on hold with a task-oriented coping style (Swann et al., 2016). Furthermore, the vulnerable survivors remained in a state of shock longer, and therefore needed support from others (Swann et al., 2016). These survivors were found unable to contribute to the immediate relief efforts as opposed to the survivors that were described as mentally tough (Swann et al., 2016).

Avoidant coping styles have been associated with more acute stress reactions (Eid, Johnsen, & Thayer, 2001), with increased stress symptoms over time (Johnsen, Laberg, & Eid, 1998), with increased risk of sensitization (Johnsen, Eid, Laberg, & Thayer, 2002), and with increased alcohol consumption and reduced well-being (Johnsen et al., 1998) in three different Norwegian military disaster studies (i.e., survivors of shipwreck and avalanche disasters).

However, the most interesting for individuals that experience such symptoms, as well as for the health personnel treating them, are how these symptoms impact daily life in both short term and in the long run (Cerdá, Borfelois, & Galea et al., 2013; Levitt, Malta, Martin, Davis, & Cloitre, 2007, Maguen, Stalnaker, & McCaslin et al., 2009; Malta, Levitt, Martin, Davis, & Cloitre, 2009, Shea, Vujanovic, & Manfield et al., 2010; Westphal et al., 2011). Adaptive coping strategies (i.e., active coping skills) are considered as a factor promoting resilience (Iacoviello & Charney, 2014). Such skills incorporate behavioural and cognitive components (Iacoviello & Charney, 2014). However, research has reported that coping strategies can be learned and thus be trained (Rice & Liu, 2016). The Australian Army had such a training program, which was designed to facilitate the use of adaptive coping strategies (Cohn & Pakenham, 2008). This study reported that the intervention group had less use of negative coping strategies, less psychological distress, and more positive states of mind than the control group (Cohn & Pakenham, 2008). Knowledge about how survivors cope with different consequences and symptoms after natural disasters as an avalanche, and the impact of daily life, might help health personnel and the institutional system (here: military organization) to identify and improve targets of intervention. Such knowledge can contribute to reduce the lasting disabling consequences following natural disasters. Therefore, gaining knowledge on this topic is important, particularly to explore the phenomena of daily living.

The aim of the study was to explore and describe experiences of daily life after having experienced an avalanche three decades ago.

The specific research questions were:

- What are the survivors’ experience of their health condition and daily life?
- How do the survivors cope in daily life?

## Context

The background scenario for this study was a military NATO winter exercise called Anchor express. A few minutes past 1:00 p.m., 5 March 1986, an avalanche struck a platoon of 31 young soldiers from an engineering corps at Vassdalen, Norway, and left 16 dead and 15 survivors (Herlofsen, 1994). This study is part of a longitudinal follow-up study among a group of exposed and unexposed soldiers, 30 years post-disaster (Bakker et al., 2019). The participants in our paper are only the directly exposed survivors, not their indirectly exposed peers. However, we know from a recent quantitative study that six out of twelve (6/12) exposed survivors in our study reported present sleep quality problems above cut-off (Bakker et al., 2019), and had most likely greater odds of hyperarousal symptoms during the whole follow-up period compared to those without sleep quality problems 30 years post-disaster (Bakker et al., 2019). Further, this study also reported that eight out of twelve (8/12) survivors had experienced more than one potentially traumatic events (PTE) in their lifetime, three out of twelve (3/12) were on disability, and, lastly, eight out of twelve (8/12) survivors answered that the disaster has affected them negatively both mentally and physically (Bakker et al., 2019). Furthermore, another recent study of our survivors (Bakker et al., 2019) measured posttraumatic stress, distress, and anxiety symptoms at four-time points: 4 days (T1), 30 days (T2), 375 days (T3), and 30 years post-disaster (T4). Findings showed that the mean values across all measures decreased over the first year post-disaster (T1-T3) (Bakker et al., 2019). These results are mostly in line with previous short-term studies investigating TEs (Arnberg, Eriksson, & Hultman et al., 2011; Bøe et al., 2011; Eid, 2003; Koren, Arnon, & Klein, 1999; Sundin & Horowitz, 2003; Thordardottir et al., 2015). However, all latter mentioned mean values increased again from T3 to T4 (Bakker et al., 2019). Measures of posttraumatic stress and distress symptoms at 30 years’ post-disaster (T4) were above all previous mean values (i.e., T1-T3) (Bakker et al., 2019). These findings, in turn, are not in line with long-term studies on survivors (Arnberg et al., 2011; Bøe et al., 2011; Green et al., 1990; Holgersen, Klöckner, Bøe, Weisaeth, & Holen, 2011; Hull, Alexander, & Klein, 2002; Lazaratou et al., 2008; Lundin & Jansson, 2007; Neria et al., 2008; Norris et al., 2002, Thordardottir et al., 2015). Caseness above cut-off point from the study, indicates need of psychological referral for (i) posttraumatic stress symptoms (PTS) in five out of twelve, (ii) distress symptoms in six out of twelve, and (iii) anxiety symptoms none of the twelve at T4 (Bakker et al., 2019). Lastly, previous studies by Rostrup, Gilbert, & Stalsberg (1989) and Stalsberg et al. (1989) reported a considerable proportion of physical injuries among our participants directly after the avalanche. For additional new data regarding subjective clinical variables, i.e., variables of alcohol consumption, see Table I.

**Table I.** Subjective clinical variables of soldiers exposed to the avalanche at Vassdalen in 1986–30 years post-disaster.

	Exposed (N = 12)
**Age**	
Mean age—30 years post-disaster	52.4
Mean age at time of avalanche	20.5
	**n/N**
**Compared to the alcohol consumption pre-disaster, how is your alcohol consumption after the disaster?:**	
Six months post-disaster:* Lower than before Same as before Higher than before Much higher than before *Six to twelve months post-disaster*: Lower than before Same as before Higher than before Much higher than before Today—30 years post-disaster:* Lower than before Same as before Higher than before Much higher than before	1/12 6/12 3/12 1/12 0/12 6/12 3/12 3/12 5/12 6/12 0/12 0/12

* Missing value = one out of twelve

## Method

### Design

This study had an explorative design, based on retrospective, qualitative interviews (Graneheim & Lundman, 2004) to provide knowledge about experiences of daily life after having experienced an avalanche three decades ago.

The interviews were analysed by means of inductive qualitative content analysis as described by Graneheim and Lundman (2004). Content analysis is a method of analysing written or verbal communication in a systematic way (Graneheim & Lundman, 2004). Further, this method is useful in analyses of a group’s or person’s reflections, attitudes, and experiences (Graneheim & Lundman, 2004).

### Participants

Recruitment took place between August 2016 and August 2017. All the exposed avalanche survivors were alive and traceable. In total, 15 survivors were contacted by postal mail, in accordance with the sampling strategy. Three survivors refused participation, yielding 12 interviewed survivors. The survivors’ mean age at time of the avalanche was 20.5 years, and mean age at the interviews was 52.4 years. For further description of the characteristics of the exposed soldiers see previously published research (Bakker et al., 2019).

### Data collection

This qualitative study uses in-depth interviews with broad open-ended questions. The interviews were guided by a thematic interview guide (see Table II). In order to discuss the feasibility of conducting this study, we first gathered possible participants for a joint meeting. We recognized that the discussions tended to veer towards irrelevant issues and the dialogue seemed to suffer from the dominance of some participants. Based on these observations and to gain more detailed information from each participant, we decided to use individual interviews. Individual interviews may offer insight into the participants’ personal feelings, thoughts and world view (Knodel, 1993; Morgan, Scannell, & Krueger, 1998).

**Table II.** Broad open-ended interview guide.

*Please describe how you have coped/managed to live with the avalanche disaster in daily life afterward?* ∘ Follow-up questions during the interview might be e.g., that interviewer asked the survivors to talk about/deepen/describe in more detail the challenges that came up in the interview: i.e.,
▪ Can you tell me more about how often you drank alcohol aftermath?
▪ Could you describe more the sleep problems you talked about?
▪ What do you think about other conditions at work or in your private life that were stressful during the period post-disaster?
▪ How did you cope with that in your daily life?
▪ Do you have the same resources or coping strategies available today, that you think are important today, 30 years’ post-disaster?

On average interviews were 95 min in duration, ranging from 20 to 180 min. The majority of the interviews took place in hotel rooms, a few in the first author’s office and one of the interviews was held in one of the survivors’ home according to the participant’s wish. The dialogue flowed very well during the whole interview, and some of the participants confirmed that the conversation had turned out better than they had expected. All participants confirmed that they had a positive opinion of the session at the end of the interview. The first author (LPB) performed all the interviews, which were recorded as audio files, transcribed verbatim by a professional firm, and safely stored. The audio files and transcripts did not contain the names of participants, and a separate “key” with the participants’ names was created on a secure, separate drive, matching the file with the participants’ codes. The verbatim account was reviewed only by the interviewer (LPB) and by two of the co-authors (EKG and SE).

### Data analysis

The qualitative content analysis, with the search for manifest and latent meanings, was led by LPB and performed in several steps. The analysis was inspired by Graneheim and Lundman (2004). One of the co-authors (EKG) participated fully in the analysis process, in which the first step was to become acquainted with the data from the interviews without applying any theoretical perspective. Further, we discussed the actual theme and suggested descriptions (the manifest meaning) that emerged from the content analysis (Carver et al., 1994; Lazarus & Folkman, 1984; Skinner et al., 2003). The analytical process is described in four steps below.

### Description of the four analytical steps

*Step 1*: In order to catch the impression of the whole, the first author (LPB) and one of the co-authors (EKG) read closely all the transcribed interviews several times. Both researchers’ impressions of every interview were written down separately and summarized in a short text of 400–800 words, and thereafter discussed in-depth several times by the first author and co-author. An early consensus on the impressions of the interviews was established through those discussions.

*Step 2*: Each interview constituted one unit of analysis and was deconstructed into units of meaning that were condensed (LPB and EKG). This was done by focusing on staying as close as possible to the survivors’ own descriptions (self-understanding).

*Step 3*: The meaning units were further abstracted and labelled with a code (LPB and EKG). All the various codes were compared based on similarities and differences and sorted into fields of content and tentative categories (LPB and EKG). The results of step 3 were entered into a spreadsheet (see Table III). From this we were able to perform the analysis across individuals, looking for variations, differences and similarities in the descriptions (LPB and EKG). During this analysis process, three different categories emerged across the survivors, indicating similarities in attitudes and how they coped and perceived their health condition and lived their daily lives.

**Table III.** Examples of development from units of meaning to categories.

Units of meaning	Code	Category
*“I felt I acted quite appropriately then. I was also a bit proud of the way I had responded to the avalanche.”*	Proud of how I responded to the avalanche	**Consequences of processing the disaster: A comfortable life**
*“I have a pragmatic approach to the psyche anyway. I do not dig into things.”*	Pragmatic approach	**Consequences of processing the disaster: A comfortable life**
*“The accident has helped me to reflect more on what’s good and what’s bad.”*	Self-reflection	**Consequences of processing the disaster: A comfortable life**
*“I needed help to sort things out, because it was bad for my night-time sleep and my concentration at work. I contacted a health professional and made a few appointments with him, and that sorted it out.”*	Good help to being able to speak about the disaster	**A challenging, yet accomplished life**
*“I enjoy physical activity. Is that a flight and a distraction, or is it a pleasure? I’m not entirely sure, but as long as it gives me something, I do not need to have the answer to that.”*	Could physical activity be a flight or distraction?	**A challenging, yet accomplished life**
*“In the period after the avalanche I was not very keen on skiing in the winter, but I did go again a few years later.”*	Not keen on skiing, but did it anyway	**A challenging, yet accomplished life**
*“I do not like the mountains anymore. I prefer them at a distance.”*	Mountains on a distance	**A demanding life**
*“During the first year aftermath there was a lot of drinking […] I think it was to forget everything”*	Drinking to forget	**A demanding life**
*“I said nothing, or very little about it.”*	Not talking about the disaster	**A demanding life**

*Step 4*: The three different categories found in step 3 were discussed in depth. After several meetings and dialogues between the first- (LPB) and two of the co-authors (EKG and SE), the underlying, latent content of the three categories was formulated into one theme.

Examples of the development from units of meaning into codes and categories are given in Table III.

### Ethical considerations

The participants were provided written information and signed the consent form. Before and after all the interviews the participants were told that uncomfortable thoughts and feelings might arise, and that some psychological and physical reactions to the interview may occur and last for a few hours, or perhaps as long as a few days after the interview. The interviewer highlighted the fact that such reactions are normal. Furthermore, all participants who wanted professional psychiatric aid were offered support from the Institute of Military Psychiatry.

Given the rich data from qualitative interviews and the reporting of the avalanche disaster in the media, it is a possible risk of reidentification. Therefore, a decision was made to restrict the reporting of demographic characteristics of the sample to protect the participants’ privacy. Deductive disclosure, also known as internal confidentiality, occurs when the traits of groups or individuals make them identifiable in research reports (Kaiser, 2009). The study was approved by the Norwegian Regional Committee for Medical Ethics (Reference number: 2016/392), and conforms to the ethical principles for medical research on human beings set out in the declaration of Helsinki (World Medical Association, 2013).

## Findings

One main theme was identified from the content analysis: “Finding my own way of managing and dealing with life”. Further, the content analysis revealed three different categories which describe the participants’ experiences in living their daily lives during three decades post-disaster: (i) A comfortable life; (ii) A challenging, yet accomplished life; (iii) A demanding life.

### The theme

The three categories represent different ways dealing with the avalanche experience in a qualitative perspective. A comprehensive understanding of the categories was discussed in light of the aim of the research, and an overall synthesis of the categories generated revealed the comprehensive understanding and the latent meaning expressed as: “Finding my own way of managing and dealing with life”.

### Description of the three categories

#### A comfortable life

The survivors in this category described that they considered being alive as being the most important thing after the avalanche. Based on this, they described that the circumstances could have been even worse: “I’ve been quite fortunate despite the circumstances, I think.” Further they described that they could not have acted differently regarding solving the challenges directly and later on during the decades’ post-disaster: “I felt I acted quite appropriately then. I was also a bit proud of the way I responded to the avalanche.” The survivors also described how they managed to cope with living with the avalanche disaster in everyday life with no special mental problems post-disaster: “No one has any such mental disorders in our family […] so I think that is the case for me too, it is both heritage and environment then.” Further, the surviors in this category described that they did not invest much effort in negative thoughts. They grew confident from how they had been able to meet stressors that everyday life had given them so far: “I have a pragmatic approach to the psyche anyway. I do not dig into things.” Furthermore, the survivors described active attempts to how they restructured and changed their view of a stressful situation in order to see it more positively: “The accident has helped me reflect more on what is good and what is bad.” Another noted: “More positive than negative things have come out of that disaster. I have become more aware that there are things I might have learned from it. It has made me a better person.”

Further, the participants of this category described a broad variety of ways to seek support and to talk about the disaster during the three decades’ post-disaster. The participants described that they were not afraid turning to others (i.e., family, friends or co-workers) in order to gain emotional support or to talk generally about the disaster and feel comfortable with it: “I got a lot of attention. I had lots of chances to talk about what I had been part of. So I’ve probably had some therapy through that.” Further, the survivors described the cohesion to the other survivors in the platoon was important to cope with the disaster during the 30 years’ post-disaster: “I have always been looking forward to the five-year meetings. It has been a very nice group, plus that you, in a way, get to meet others who have had the same experience.” However, the surviros described a lack of support from the military system following the disaster: “I believe that the armed forces did not contribute much after the avalanche.”

#### A challenging, yet accomplished life

This category incorporates a wide range of experiences, attitudes and strategies towards the experience of living with the avalanche in daily life. The survivors described that they reacted very differently with regards to how much effort in negative thoughts and behaviour they had used on the disaster in their daily life during the three decades’ post-disaster. Survivors in this category described a wide variety of distraction techniques such as working a lot or performing physical activities. However, they described that they were not familiar with these techniques to cope with emotional or other psychological challenges post-disaster: “For a long time, I worked a lot. I wonder afterwards, if that was because I had an interesting and good job, but was it really because I needed a distraction?” Another noted: “I enjoy physical activity. Is that a flight and a distraction, or is it a pleasure? I’m not entirely sure, but as long as it gives me something I do not need to have the answer to that.”

Further, other survivors in this category described that they tried to overcome anxiety for winter activities in a period after the disaster: “In the period after the avalanche I was not very keen on skiing in the winter, but I did go again a few years later.”

Seeking support and talking about the disaster, and other daily adversities, was described at different levels in this category. This category describes all levels, from not talking about the disaster at all, to talking to others about it, and to seeking advice and help from peers, family, community or health personnel at different periods’ post-disaster. This was done differently in the years from directly after the disaster to approximately 30 years’ post-disaster: One noted “I needed help to sort things out, because it was bad for my night-time sleep and my concentration at work. I contacted a health professional and made a few appointments with him, and that sorted it out.” Another noted: “If you experience negative things, tell someone about it.” Other survivors described that they talked a lot during the first year post-disaster: “The first year after the disaster I think I talked a lot and got it out of my system.” Other survivors in this category described other ways to overcome/cope with the distress and to talk about the disaster in everyday life: “I understood early that talking about it, even though it was unpleasant, was good. The more uncomfortable, the more necessary. I have always thought so.” Others spoke about how they coped with everyday life challenges by themselves: “I’m probably not a person who actively uses the network around me. I like to get things done by myself.” Further, they also described lack of support from the armed forces both in the short and long term: “I feel a legitimate resentment for absenteeism from the armed forces post-disaster.”

#### A demanding life

The participants in the third category described, and emphasized, how they struggled to cope with everyday life after the avalanche disaster. They described symptoms of severe mental consequences (i.e., symptoms of posttraumatic stress, distress, anxiety, and sleep problems) in daily life during the three decades’ post-disaster: One noted “After the disaster they began to appear, the nightmares.” Others described anxiety and hyperousal symtpoms when thinking of similar situations as the avalanche: “I do not like the mountains anymore. I prefer them at a distance.” Another noted: “I don’t want to get into situations that remind me of the avalanche, I think about it every day.” Further the participants in this category described periods of large alcohol consumption following the disaster: “lots of alcohol followed decades post-disaster […] but I had to stop drinking.” Another noted: “during the first year aftermath there was a lot of drinking […] I think it was to forget everything.” Furthermore, description of problems with occupational functioning were only described in this category: “after the disaster I have mostly been unemployed.”

During the interviews the survivors in this category seemed to describe a limited variety of ways to talk about the disaster to other people. Most of the survivors in this category said that they did not talk to others or ask for advice or help from others to handle daily life after the disaster: “I said nothing, or very little about it”, while a very few described that they felt that they talked too much to others about the disaster: “I talked a lot to people about this. I’m sure many were tired of hearing me talking.” The participants in this category also described lack of support from the military system post-disaster: “the armed forces did very little for us.”

For the participants in the third category, the avalanche was a central concern which represented a daily challenge to live everyday life.

#### Summary of findings from a theoretical perspective

The three categories seem to represent different ways of dealing with stressors in everyday life post-disaster. “Finding my own way of managing and dealing with life” describes different ways of coping with the situation. The survivors representing the category *“A comfortable life”* tended to use strategies such as problem solving, talking about it (seeking social support), reflecting on their experiences (positive cognitive restructuring) as well as focusing on the positive aspects of their current situations. These strategies appeared to be adaptive for these participants in their contexts and improved their mental well-being. The survivors in the second category *“A challenging, yet accomplished life”* were also dealing with most of the different types of stressors in their everyday life, using adaptive coping strategies during the whole period. However, there were several descriptions of strategies that were interpreted as more maladaptive in this category than in the first category analysed, i.e., avoidant strategies such as avoidance and distraction. These strategies appeared to be more adaptive than maladaptive for these participants in their contexts. For the third category, *“A demanding life”*, the survivors tended to use strategies such as avoidance and distraction in everyday life post-disaster. These strategies appeared to be maladaptive for these participants in their context and resulted in impaired mental well-being.

## Discussion

This study aims to explore and describe the experience of survivors’ health and how they cope with everyday life after an avalanche disaster during three decades post-disaster.

In an early analytic stage, we saw that our three categories were compatible with coping theories, and we decided to discuss the categories in relation to the coping strategies provided by Skinner et al. (2003) five core categories of coping. Our purpose for applying these coping strategies was to use well-supported and known domains from literature on coping that covered a diversity of behaviours and thoughts (Skinner et al., 2003). However, other theories and literature will be applied to cover a broader perspective to shed light on the topics.

### “A comfortable life”

The findings show that the coping strategies described in the first category seemed to result in greater well-being and functioning during the three decades’ post-disaster, compared to the other categories. The survivors described how they managed to cope with the avalanche disaster in everyday life by using coping strategies interpreted as adaptive. The first category seemed primarily to be consistent with three of Skinner et al. (2003) core categories of coping: (i) positive cognitive restructuring, (ii) problem solving and (iii) seeking social support. The avalanche disaster did not seem to be of great importance. Furthermore, the participants did not invest much effort in negative thoughts regarding the disaster, and mainly used a form of positive cognitive restructuring to actively change their thinking around the stressful situation in order to see it more positively. The description of coping strategies used in the first category may fall under what the research literature in the field refers to as problem-solving and active emotional coping strategies (Skinner et al., 2003). In quantitative studies, these types of coping strategies, especially problem solving and seeking support, contribute to better and healthier functioning (Cherry et al., 2017; Littleton et al., 2007; Zimmer-Gembeck & Skinner, 2008). This is also consistent with qualitative studies conducted on survivors after natural disasters, where seeking support, problem-solving, and seeking meaning are the most cited coping strategies contributing to better and healthier functioning (Ekanayake et al., 2013; Ibañez et al., 2004; Rajkumar et al., 2008). Some of the survivors in this category described that they had a “pragmatic approach” to the psyche and did not “dig into things”. One could ask if this approach is maladaptive, an avoidance or distraction in everyday life to “forget” about the disaster’s impact?

This kind of approach seems to allow the first category of survivors an assimilation and acceptance of the traumatic experience into life and provide opportunities for recovery and growth. This is in accordance with previous research that described the attempt to forget as a kind of cognitive flexibility. This would enable the survivors to reappraise the perception and experience of a TE, providing opportunities for growth and recovery (Cherewick et al., 2015; Iacoviello & Charney, 2014). On the other hand, this may constitute a resilient behaviour: an expression of a personality trait referred as “mental toughness” (Hardy, Bell, & Beattie, 2014), which is also considered as an ability to cope with or handle pressure, stress or adversity (Goldberg, 1998; Gould et al., 1987; Jones, 2002; Williams, 1988). Furthermore, mental toughness might also reflect that these participants felt that they were “acting quite appropriately” during and after the disaster. However, the participants are former military personnel who presumably have high levels of mental toughness and we can assume that they have traits or abilities to cope with adversity. These latter reflections above are consistent with a recent study of survivors of an avalanche, sheding light on the positive role mental toughness has on coping during and shortly after a natural disaster (Swann et al., 2016). Another quantitative study among athletes, reported that higher mental toughness is associated with less use of avoidant/emotional coping and a greater use of problem-solving coping strategies (Nicholls, Levy, & Polman et al., 2011). On the other hand, it might be that the participants in this category are more resilient and have a positive adaption to change than particpants in the other categories. It seems like they act and create their own resilience by using adaptive coping strategies such as problem-solving, cognitive restructuring and seeking social support. This is in accordance with literature on the field of resilience, for instance Iacoviello and Charney, (2014, p. 3) highlighting that: “Resilient individuals use active rather than passive coping skills; they act and create their own resilience.” The survivours interviewed seemed to describe a resilient or resistant pattern regarding trauma-related psychopathology (Bonanno, 2004; Norris et al., 2009). However, in disaster literature, adaptive coping styles have been found to be associated with better and healthier functioning, less psychological distress and better health (Ekanayake et al., 2013; Ibañez et al., 2004; Rajkumar et al., 2008; Xu & He, 2012).

Although it seems like the coping strategies described by the participants in the first category influencing their mental health outcomes for the better, compared to the participants in the other categories. Therefore, we have an understanding of the participants using adaptive coping styles such as cognitive restructuring, problem-solving and seeking social support, may have fewer mental health problems. This is consistent with previous research stating that individuals influence their mental health for the better regarding to their ways of coping (Freedy, Saladin, Kilpatrick, Resnick, & Saunders, 1994; North, Spitznagel, & Smith, 2001). However, this could also be in accordance with a natural disaster study which reported that men who apt to adapt adaptive coping strategies may have fewer negative psychological outcomes (Xu & He, 2012).

The latter descriptions and findings might also be in accordance with two recent quantitative studies of our sample (Bakker et al., 2019, 2019) reporting PTSD-symptoms and sleep quality problems below cut-off point for some of the participants. This would indicate no need of psychological referral in some of the survivors 30 years’ post-disaster (T4). These previously reported findings regarding our sample, might indicate that the interviewed survivors in this category might fit the reported group of survivors with less severe psychopathology symptoms, and absence of risk factors (i.e. description of no prior mental illness, to have a job, and no prior PTEs before or after the avalanche (Bakker et al., 2019)).

Literature describes that resilient individuals seek acknowledgement of social support (Iacoviello & Charney, 2014). Participants from the first category described that the meetings every fifth year with the other survivor peers seem to contribute to a positive cohesion. This is in line with the literature describing the importance of contributing to considerable emotional strength for those involved in TEs (Iacoviello & Charney, 2014).

### “A challenging, yet accomplished life”

The second category of survivors described a wide range of coping strategies towards the experience of living with a severe traumatic event in everyday life compared to the other categories. The coping strategies described were interpreted as a combination of all Skinner et al. (2003) five coping strategies. Compared to the other categories, the survivors in this category described a much wider view of the impact of the disaster, and whether they had experienced any challenges in their daily life during the three decades’ post-disaster. The survivors described that challenges may still exist. However, compared with participants from the third category, they described a greater acceptance and less use of the maladaptive coping strategies in everyday life, which might interfere negatively with mental health. On the other hand, it seems that these survivors are more negatively affected in daily life by the disaster, compared with the first category. Nevertheless, it is difficult to argue that the few adaptive coping strategies described in the first category are more preferable than the combination of coping strategies used in the second category. This is supported by both Skinner and Zimmer-Gembeck (2007) and Zimmer-Gembeck and Locke (2007), who argue that the most adaptive strategy is to be able to use a wide range of coping strategies and being able to employ them when needed. This might be the case for these participants as they described adapting to their environments well, being able to use a broad range of coping strategies and employ/use them when needed. This is in accordance with literature describing coping, and resilience (Kim-Cohen & Turkewitz, 2012), as a dynamic process that fluctuates over time in response to changing appraisals and demands of the situation (Afshar et al., 2015; Diehl et al., 2014; Moos et al., 2003). Even though survivors in the second category used more strategies described as maladaptive (e.g., avoidance and distraction) compared with the first category, it seems like the survivors found that distracting alone and keeping busy with exercise or work could be a successful way of dealing with the disaster in everyday life. These findings are compatible with what Ekanayake et al. (2013) found in their qualitative study of survivors after a tsunami describing that keeping busy was a successful way of dealing with stress (Ekanayake et al., 2013).

The participants in the second category described a broad variety of ways to seek support (i.e., family, peers, community or health personnel). This seemed to make a positive influence on their emotions, thinking about themselves and had a protective impact on negative mental health outcomes. These observations and descriptions are consistent with previous research suggesting that social support influence individuals’ own thinking about themselves and protect against negative psychological outcomes of trauma (Panzarella, Alloy, & Whitehouse, 2006). Although the fact that this category describes little use of maladaptive coping strategies and symptoms associated with psychopathology compared to the first category, we observed that the participants in the second category described higher levels of psychopathological symptoms and challenges than the survivors in the first category, but less than in the third category. These observations might be consistent with studies claiming that many survivors of TEs never will experience, or be given an opportunity to report all the symptoms for a full diagnosis of PTSD. However, having a sub-threshold or subsyndromal PTSD in periods, may impair functioning close to a fully diagnosed PTSD (Breslau, Lucia, & Davis, 2004; Norman, Stein, & Davidson, 2007; Pietrzak et al., 2012; Schnurr, Friedman, & Rosenberg, 1993).

We might interpret, from the descriptions, that the survivors in the second category might follow different patterns than the survivors in category one, regarding trauma-related psychopathology, i.e., a recovery, delayed (Bonanno, 2004; Norris et al., 2009) or a U-shaped pattern (Macleod, 1994; Port et al., 2001). A previous study of our sample may support a U-shaped pattern for our participants (Bakker et al., 2019) reporting that the time trajectories for PTS-symptoms indicates a U-shaped course for all our participants during the observed 30 years (T1-T4) (Bakker et al., 2019). Regarding the descriptions from the survivors’ daily life in the second category, the findings indicate that the U-shaped pattern may fit very well for the participants in this category compared with the two other categories.

### “A demanding life”

The descriptions and findings indicate that the few coping strategies that are applied (i.e., avoidance and distraction) by the third category of survivors, are interpreted as difficulties with coping with everyday life after the avalanche.

The disaster is described to be of central importance in this category, and the survivors describe having a lot of negative thoughts and behaviours regarding the disaster three decades’ post-disaster. Survivors in this category described that they did not seek support for advice or help from others to handle the consequences of living with the disaster. Furthermore, most of them described that they were uncomfortable talking to others about the disaster and about their lives. Such behaviour, even though it is shown as different expressions, may be in accordance with previous quantitative research of Norwegian veterans that showed barriers to seek health care for mental health problems (Johnsen & Böe, 2016). Several quantitative studies support that veterans with mental health problems do not seek health care because seeking such care may be associated with weakness (Hoge et al., 2004; Johnsen & Böe, 2016; Kim, Thomas, Wilk, Castro, & Hoge, 2010). This could be an explanation for not seeking support, advice or help.

The descriptions of using maladaptive coping strategies include the presence of several symptoms which are known to go hand in hand with PTSD-symptoms (i.e., PTS, distress, anxiety symptoms, and sleep quality problems), e.g., avoidance of situations that may remind them of the avalanche and nightmares, and further, descriptions of abuse of alcohol in periods afterwards to forget or avoid feelings around the avalanche. According to previous research in the field of coping, the coping strategies described by the third category refer to types of coping strategies that have been found to be associated with impaired functioning, poor health and psychological distress (i.e., avoidance and distraction) (Bei et al., 2013; Cherry et al., 2017; Littleton et al., 2007; Schnider et al., 2007; Zimmer-Gembeck & Skinner, 2008). Furthermore, Horowitz (1986) has described that the more intense the TEs are, more likely survivors will have stress reactions involving avoidance and distraction. In such situations, avoidance and distraction can be considered adaptive by reducing stress in a short period. However, these strategies are considered positive for short-term stressors, and negative if used long term regarding the traumatic situation (Gibbs, 1989; Suls & Fletcher, 1985). The participants in the third category are observed to be describing just such use of long-term trauma avoidance and distraction strategies that might have negative impact on their mental health outcome in the long-run. Further, all PTSD-symptoms (i.e., PTS, distress and sleep problems) described in the third category are consistent with two recent-published studies on our sample (Bakker et al., 2019, 2019). In these two studies the participants reported considerable symptom burden above cut-off point, e.g., in need of psychological referral, respectively five out of twelve above cut-off for PTS symptoms, six out of twelve above for distress symptoms, and six out of twelve above cut-off regarding sleep quality problems (Bakker et al., 2019). Furthermore, the third category also corresponds with previous reported findings from the sample of risk factors that may predict and increase vulnerability to develop a mental health disorder post-disaster, e.g., survivors on disability (Bakker et al., 2019), survivors with grad school or less (Bakker et al., 2019), survivors that reported more PTEs than just the avalance (Bakker et al., 2019) and so on and so forth.

Additional subjective clinical variable reported in our paper, could indicate that some of our participants might abuse alcohol today 30 years’ post-disaster, see Table I. This subjective clinical variable indicates that in total six out of twelve of our participants reports “higher” or “much higher” alcohol consumption six to twelve months post-disaster. These latter results could coincide with the descriptions in the third category that described different challenges regarding alcohol during the thirty years’ post-disaster. However, it is important to emphasize that some of the participants in the third category described stopping abusing alcohol after a year or decades after disaster.

Furthermore, alcohol intake is associated with possible mental disorders in several studies (Hougsnæs, Bøe, Dahl, & Reichelt, 2017; North, 2016). This could also coincide with the description of psychopathology symptoms in the third category (i.e., PTS, distress and sleep problems). This latter observation are in accordance with a quantitative study of Norwegian veterans which showed that current alcohol intake was significantly associated with probable mental disorders (Hougsnæs et al., 2017). The alcohol abuse described in the third category may be interpreted as an avoidant coping style to handle daily life. This interpretation of the described alcohol consumption can, further, be in accordance with another quantitative study of American veterans that claims that the strongest factor associated with alcohol abuse in returning soldiers is an avoidant coping style (Bartone et al., 2015). Other quantitative studies have also highlighted the connection between PTSD and drinking behaviour as “drinking to cope” (Lehavot, Stappenbeck, Luterek, Kaysen, & Simpson, 2014), drinking to regulate emotions (Cooper, Frone, Russell, & Mudar, 1995), and the use of alcohol to regulate negative effects in the absence of more adaptive emotional coping strategies (Veilleux, Skinner, Reese, & Shaver, 2014); these connections may be present in our study too, and especially for participants in the third category that describes a problematic alcohol consumption.

Two previous quantitative studies of Norwegian soldiers (Eid et al., 2001; Johnsen et al., 1998) may also support that avoidant coping strategies could coincide with the descriptions in the third category (e.g., participants’ description of avoidance of situations that may remind them of the avalanche, and description of alcohol as a mean to forget). These two studies describe avoiding coping styles to be associated with more acute stress reactions (Eid et al., 2001) and related to an increase of stress symptoms over time, increased alcohol consumption and low well-being in the soldiers (Johnsen et al., 1998). From all the observations and descriptions we made from the survivors in the third category it might seem that they describe to follow a different pattern than the survivors in the first and second category, regarding trauma-related psychopathology. It could seem that the participants in the third category, as a whole, give a description of following a pattern described as a chronic pattern, i.e., pattern where trauma-related psychopathology symptoms tend to persist across time (Bonanno, 2004), more than a U-shaped pattern as in the second category.

Lastly, all participants in our study described a lack of support from the military system post-disaster. These descriptions of lack of support might have affected the participants in our three categories differently since we know from literature that our action towards stressors and TEs takes place in a context of interaction with other individuals, cultures, available resources, communities, and organizations (Iacoviello & Charney, 2014; Sherrieb, Norris, & Galea, 2010; Southwick et al., 2014; Walsh, 2006) (e.g., military as an organization), and that we have to see beyond just individual factors that may promote coping and resilience (Docena, 2015; Kirmayer et al., 2011; Kruse et al., 2017; Meredith et al., 2011; Rice & Liu, 2016; Southwick et al., 2014). Nevertheless, our third category is described as a category which has low-seeking of support and high levels of psychopathology symptoms. The third category most likely may have been further adversely affected by a non-supportive military organization. A previous study of veterans returning from wars support these assumptions (Tsai, Harpaz-Rotem, Pietrzak, & Southwick, 2012). This latter study found that less social support from community/organization and lower availability of secure relationships mediated the association between PTSD and poor social functioning.

The descriptions of experiences in all three categories in our study illustrate that it might be a broad variation in how impact of trauma experienced earlier in life might affect the coping strategies in daily life later on.

We know from previous studies that TEs are common and the probability of TEs to occur is high, seen in a lifespan perspective. It is not a question of if, but when it is going to happen! That is why we must prepare individuals for exposure, so this do not happen to be a shock. Additionally, we have to enhance resilience through strengthening adaptive coping strategies to deal with adversity. This is even more important to the individuals who are considered less resilient, because not everyone who uses coping strategies is considered resilient (Rice & Liu, 2016). And we know from literature that active coping strategies mediate promoting resilience (Iacoviello & Charney, 2014). Further, it is important to emphasize that research has found that coping strategies can be learned and thus can be trained (Cohn & Pakenham, 2008; Rice & Liu, 2016). Therefore, insight into coping strategies may provide a guide for appropriate interventions for survivors in dealing with TEs in the short and long run, e.g., through building coping and resilience programmes on an individual, organization and community level.

### Strengths and limitations

The coping strategy findings presented in our study are highly context-specific, and might present an oversimplification of the survivors’ coping with the disaster in their daily lives; other important experiences, not identified in the interviews, may have influenced the way they coped with the disaster. However, the present study yields rare insight into a trauma area where hardly any study supplies survivors’ descriptions. This is an advantage of using a qualitative method.

We used trauma, stress and coping strategy theories in the interpretations of the findings. It is important to emphasize that the relationships between TEs, negative health outcome, reduced quality of life and coping strategies are complex and still not fully understood (Araya, Chotai, & Komproe et al., 2007; Skinner et al., 2003). Further, it is also important to emphasize that research literature argues that rigid reliance on just a few coping strategies may indicate problems in managing stress and maladaptation (Zimmer-Gembeck & Skinner, 2008). Furthermore, there might be several more descriptions of patterns that have been reported in the literature regarding the course of PTSD-symptoms and trauma-related psychopathology than our study have chosen to use, e.g., cyclical and quadratic patterns (Davidson & McFarlane, 2006; Norris et al., 2002). Nevertheless, we consider the patterns described and chosen in our study to cover the most cited patterns in literature.

Our purpose in applying the five-fold coping strategies developed by Skinner et al. (2003) was to use well-supported domains from literature on coping that cover a broad variety of behaviours and thoughts. However, the five coping strategies by Skinner et al. (2003) are nuanced, and coping strategies may overlap in our material. A specific mindset or coping strategy may serve one or several purposes (Seguin, Lewis, Razmadze, Amirejibi, & Roberts, 2017), e.g., working may represent both a problem-solving and a distraction strategy/activity for the survivors in our study. Another limitation in our study might be that the survivors in all three categories seem to use the same approach of not talking about or thinking about the disaster. This may seem contradictory. They used different coping strategies to solve this which had different impact on the survivors’ well-being (e.g., the first category used positive cognitive restructuring, the third category avoidant coping strategies to approach this). However, these latter differences described may be the result of the survivors’ different personality traits (e.g., the survivors in the first and second category seem to have more adaptive coping style traits compared with the survivors in the third category). Nisa and Rizvi, 2017, p. 437) emphasizes that personality traits may influence the effectiveness of coping strategies, with strategies that are beneficial for some individuals being less effective, or even directly harmful, for those with different personality traits (Bolger, Zuckerman, & Geen, 1995; DeLongis & Holtzman, 2005; Nisa & Rizvi, 2017). This might be the case in our study too. Nevertheless, we cannot conclude that thinking/talking about or not thinking/talking about the disaster is an effective or ineffective approach to cope in general from our study. It has to be evaluated and observed in the context of the individuals interviewed.

Other theories and angles might have given different descriptions and outcome (e.g., other coping, resilience, personality trait and trauma theories). Further, another limitation could be that mental toughness has been examined within a traditional team sport setting (Cook, Crust, Littlewood, Nesti, & Allen-Collinson, 2014) and among high-altitude mountaineers (Crust, Swann, & Allen-Collinson, 2016), as in the Mount Everest study (Swann et al., 2016). The fact that mental toughness primarily is used to study sport athletes and high-altitude mountaineers may potentially provide too narrow view of the construct, and may leave limitations regarding transferring the findings to our sample of avalanche survivors. However, a strength in our study might be that we consider our sample basically selected to have relatively high levels of mental toughness ahead of the military service. We have no existing data related to our sample regarding personality traits, which could have given us some indications of traits that could be associated with adaptive (e.g., trait as extraversion) or maladaptive (e.g., trait as neuroticism) coping strategies. Although, we support findings from studies highlighting that coping may generally be affected by personality traits (Connor-Smith, Flachsbart, & Carver, 2007). Further limitations regarding comparison with other studies could be that the Mount Everest study (Swann et al., 2016) was conducted short time after the disaster and did not perform follow-up in a long-term perspective.

Furthermore, another limitation might be that we have compared findings from previous quantitative studies of the same sample as in our study, and drawn up associations of these finding to this present qualitative study. These previous quantitative studies (Bakker et al., 2019, 2019) relies on self-report rather than physical examinations and diagnostic tools. However, a strength in our study is that this is the same participants that completed the interviews short time after the survey (Bakker et al., 2019, 2019), at T4.

Our interview guide was designed for broad, open questions and emphasized daily living. The strength of this approach was that it enabled easy communication.

The findings described in this paper are based on one-time interviews, 30 years’ post-disaster. This may have reduced the depth of the discussions compared to having performed repeated interviews during the whole follow-up period. It is important to emphasize that we have to consider that the survivors’ experience of the traumatic event may vary in intensity throughout the 30 years post-disaster, and that coping is a dynamic process that also may vary over time as the survivors adapt to difficult life events (Carver et al., 1994; Cherry et al., 2017). This is compatible with what most other researchers suggest (Carver et al., 1994; Cherry et al., 2017). Further, we have to take into consideration recall bias.

With regard to reflexivity, the interviewer is a military officer and a registered nurse, and a survivor of a severe natural disaster. Further, throughout the whole analysis process, the authors emphasized reflexivity, in particular considering our backgrounds and the possible influence of the preunderstanding on the interpretation of data (Finlay, 2003).

We have presented the data with limited illustrative quotes, due to ethical considerations, because we had to reduce the potential for identifying the participants. However, the three categories are closely described, and the analysis process well documented.

Only males are included, which might be a limitation regarding the transferability of the findings. Nevertheless, a strength of this study may be that the group is homogeneous (in terms of type of trauma, age, sex, and time since trauma). However, the purpose of qualitative studies is not to generalize, but to shed light on a topic and gain in-depth knowledge from the participants (Polit & Beck, 2017). Further, in this study, we have interviewed almost all of the survivors (12/15) of the avalanche disaster at Vassdalen 30 years’ post-disaster, and the high degree of saturation in the findings may indicate that key points were well-covered.

## Conclusion

The survivors’ experiences of living their daily lives during the three decades’ post-disaster after an avalanche can be concluded in “Finding my own way of managing and dealing with life”. The survivors have different ways and ranges of coping strategies for dealing with their daily lives during the three decades’ post-disaster. Some of the survivors’ experience “A comfortable life” with a greater degree of successful coping with the disaster in daily life and seemed to have a balanced life situation. They had more or less left the avalanche behind them and looked forward more than backwards. Other survivors experience “A challenging, yet accomplished life”, where they tended to hold on to their traumatic experience, but nevertheless continued with daily life. The third way of the survivors’ experiences was “A demanding life”, which influenced the way they live with the disaster in daily life. The survivors with “A demanding life” seem to use maladaptive coping strategies interpreted as avoidance and distraction.

This paper increase insight into the consequences of adaptive and maladaptive coping strategies in a sample of avalanche survivors. Knowledge about how the survivors coped with different consequences after the avalanche, and the impact of their daily life, might help survivors, health personnel and the military system to be able to generate hypotheses for further studies and identify intervention, such as to build coping and resilience programs on an individual, organization and community level.

## Data Availability

The raw data is confidential and cannot readily be shared. Data may be shared with researchers obtaining permission from the Norwegian Regional Committee for Medical Ethics and Norwegian Armed Forces Joint Medical Services, Institute of Military Psychiatry. After permission has been obtained, data can be made available from The Norwegian Armed Forces Joint Medical Services, Institute of Military Psychiatry, contact Lars-Petter Bakker: lpbakker@mil.no

## Appendix 1.Overview of different course and trajectories of psychopathology aftermath (Bonanno, 2004; Macleod, 1994; Norris et al., 2009; Port et al., 2001)

**Table:**

Patterns and Trajectories	Definition of patterns
**Resistance** (Norris et al., 2009)	Is defined as experiencing no symptoms of mental illness or only mild symptoms post-disaster.
**Resilience** (Norris et al., 2009, Bonanno, 2004)	Pattern where symptoms are transiting and do not cause reduced psychosocial functioning following exposure to a TE.
**Recovery** (Norris et al., 2009, Bonanno, 2004)	Pattern where symptoms are prominent following exposure to a TE, and shows gradual improvement with time.
**Chronic** (Norris et al., 2009, Bonanno, 2004)	Pattern where symptoms tend to persist across time. This course is only found in relative small proportion of survivors of a TE.
**Delayed** (Bonanno, 2004)	Pattern where the symptoms are not very severe or prominent during the first 6 months following exposure to a TE, but tend to increase later (late-onset).
**U-Shaped** (Macleod, 1994, Port et al., 2001)	Pattern where there is high levels of negative mental health symptoms immediately after trauma, then declining during the years of work life but possibly returning as the survivors cope with age-related issues and transition into retirement.

## Appendix 2.Description of the five-fold coping strategies according to Skinner et al. (2003)

**Table:**

Coping strategies (1–5)	Definition
**1. Problem solving**	This domain includes categories of Cognitive Decision Making (i.e., Strategizing and Planning), logical analysis of a problem, instrumental action towards a problem, persistence, effort and determination.
**2. Seeking social support**	This domain includes a wide array of targets of support such as family, friends, peers, professionals, religious figures and/or others to solicit help, contact, advice, comfort, and/or instrumental help such as money or goods.
**3. Avoidance**	This domain includes efforts to stay away and/or disengage from stressful transaction/situation (mentally and/or physically). Includes denial, avoidant actions, cognitive avoidance, and engaging in wishful thinking.
**4. Distraction**	This domain refers to different active attempts to deal with a stressful situation. Distraction includes a broad variety of alternative activities where the persons engage in pleasurable activities, such as reading, hobbies, watching television, exercising, seeing friends, working, and substance abuse.
**5. Positive cognitive restructuring**	This domain refers to active attempts to change one’s view of a stressful situation in order to see it in a more positive light. Here the individuals focus on the positive rather than the negative by positive thinking, optimism, and minimization of negative consequences or distress.
